# Mental health of junior college students in China during COVID-19 school lockdown: Findings of on-line cross-sectional survey

**DOI:** 10.1097/MD.0000000000036808

**Published:** 2023-12-29

**Authors:** Feng Li, Jing Wang, Jiu Chen, Qian Chen, Junxia Wang, Maoxue Wang, Shouliang Ma, Bing Zhang, Wenxia Hu

**Affiliations:** a Nanjing Drum Tower Hospital Clinical College of Traditional Chinese and Western Medicine，Nanjing University of Chinese Medicine; b Department of Neurology, Lu’an Hospital of Anhui Medical University, Lu’an People’s Hospital of An Hui, Province, Lu’an, China; c Graduate School of Bengbu Medical College, Bengbu, China; d Department of Radiology, The Affiliated Drum Tower Hospital of Nanjing University Medical School, Nanjing, China; e Institute of Medical Imaging and Artificial Intelligence, Nanjing University, Nanjing, China; f Medical Imaging Center, Affiliated Drum Tower Hospital, Medical School of Nanjing University, Nanjing, China; g The First Affiliated Hospital of Anhui University of Traditional Chinese Medicine, Hefei, China; h Jiangsu Key Laboratory of Molecular Medicine, Nanjing, China; i Institute of Brain Science, Nanjing University, Nanjing, China.

**Keywords:** anxiety, COVID-19, depression, junior college students, sleep disorder

## Abstract

During the COVID-19 pandemic, junior students who had recently entered university may have experienced particular difficulties. This study aimed to investigate the incidence of anxiety, depression, and sleep status among junior college students during school closure. Junior college students from 3colleges in Anhui Province participated in this study from 6th to 20th April, 2022. The students’ data were collected using a designed online questionnaire developed on the “Wen juan xing” website and submitted via cell phone. Ordinal logistic regression analysis indicated that female sex was an independent risk factor for increased anxiety, depression, and insomnia (anxiety: OR 1.503, 95% CI 1.191–1.897; depression: OR 1.14, 95% CI 1.023–1.270; ISI OR 2.052, 95% CI 1.646–2.559). Notably, medical specialty was an independent risk factor for depression and anxiety (anxiety: OR 1.367, 95% CI 1.078–1.734; depression: OR 1.289, 95% CI 1.148–1.448). Moreover, being a freshman was a risk factor for increased depression and insomnia (depression: OR 1.036,95% CI 0.931–1.153; insomnia: (OR 1.157,95% CI 0.961–1.394). The findings indicate that a considerable portion of junior college students experienced psychological problems due to lockdowns during the COVID-19 pandemic.

## 1. Introduction

At the end of December 2019, the novel coronavirus disease (COVID-19) was first reported in Wuhan, Hubei Province, China. It then spreads rapidly across cities, provinces, and many other countries. An increasing number of infections and deaths has caused panic worldwide. COVID-19 was declared an international public health emergency by the World Health Organization (WHO) on January 30, 2020.^[1,2]^ The COVID-19 pandemic has produced negative emotions in many people. Previous studies have found that rates of anxiety, depression, loneliness, and even suicidal tendencies were – 2 to 3 times higher than usual during the COVID-19 pandemic^.[1,3–7]^ Uniquely, the city of Lu’an was relatively safe, with no large-scale spread of COVID-19 or subsequent deaths and minimal impact on daily life. However, the Omicron mutation of COVID-19^[8]^ has had a large impact on the city in the spring of 2022. When a person is sick, all residents of the city are affected because the disease is highly contagious and can spread quickly. In only half a month, the number of patients increased to hundreds. In light of the COVID-19 outbreak, the Lu’an government undertook a range of measures, including school closures, traffic restrictions, and business shutdowns. Regrettably, these actions have led to the predicament that college students are stranded at school, which has engendered diverse adverse psychological consequences, primarily due to the students’ adolescent status, confinement within confined areas, and their inability to attend classes or engage in social interactions. Previous studies have reported that domestic and foreign college students experience different degrees of mental health problems during the pandemic.^[9–14]^ There are few relevant studies on college students in lower grades, especially those who have just transitioned from high school to university.^[7,14,15]^ For such students, this was the first time that a new term had started with no face-to-face interactions, and all classes were conducted online. This study aimed to understand the mental health and sleep status of freshmen and sophomore students who remained in school during the COVID-19 pandemic to provide a basis for effective psychological counseling.

## 2. Materials and methods

### 2.1. Participants

The participants in this study were mainly Grade1 and Grade 2 students in the lower grades of 3 colleges and universities who remained on campus during the COVID-19 lockdowns in Lu’an city, Anhui province. Participants with a history of mental illness and those who were unwilling to participate were excluded. The study was approved by the Medical Research Ethics Committee of Lu’an People Hospital of Anhui Province (2022LLK012). All the participants provided written informed consent. All the participants were free to withdraw from the study at any time. Ultimately, 6905 valid questionnaires were obtained.

### 2.2. Questionnaire

We distributed the questionnaire to schools via the online “Wen juan xing” platform. The schools then released questionnaires for each student group. The students completed the questionnaires according to their actual situation. The survey consisted of 2 main parts. The first part collected participants’ gender, age, major, and grade. The second part investigated the participants’ mental health during the pandemic, including sleep, anxiety, and depression. The Self-Rating Anxiety Scale (SAS) developed by Zung^[16–18]^ was used to screen for generalized anxiety and symptom severity. The SAS is a 20-item self-report measure of anxiety levels based on 4 groups of symptoms: cognitive, autonomic, motor, and central nervous systems. It is a convenient clinical tool for the analysis of subjective symptoms. The items are scored using a 4-point scale as follows: “1” rarely; “2” some of the time; “3” a good part of the time; and “4” most of the time. Some questions were worded negatively to avoid the problem of setting responses. The scores of the 20 items were summed to obtain the total score, which was then multiplied by 1.25 to obtain the integer part and then standardized. A higher score indicates more severe anxiety symptoms. According to SAS norms for the Chinese population, a total anxiety score < 50 is considered normal, 50 to 59 indicates mild anxiety levels, 60 to 69 indicates moderate anxiety levels, and ≥ 70 indicates severe anxiety levels. Depression was assessed using the 20-item Self-Rating Depression Scale (SDS) developed by Zung.^[17,19]^ It is widely used in outpatient screening, emotional state assessments, and scientific research. The forward-scored questions A, B, C, and D were scored as 1, 2, 3, and 4, respectively. The reverse-scored questions were scored as 4, 3, 2, or 1. The scores of the 20 items were summed to obtain a total score. The standard score is equal to the integer part of the total score multiplied by 1.25. According to the SDS norm for the Chinese population, the cutoff value was 53 points, with scores of 53 to 62 indicating mild depression, 63 to 72 indicating moderate depression, and ≥ 73 indicating severe depression. Insomnia was assessed using the Insomnia Severity Index (ISI) by Moran et al,^[20]^ which is one of the most widely used insomnia assessment scales in clinical practice. The ISI includes 7 items that assess the nature and symptoms of sleep disorders in the subjects. Each question is scored from 0 to 4, resulting in a total score of 0 to 28:0–7, indicating insomnia without clinical significance; 8 to 14 indicates subclinical insomnia; 15 to 21 indicates moderate clinical insomnia; 22 to 28 indicates severe clinical insomnia.

### 2.3. Statistical analysis

The data were exported from “Wen juan xing” system and analyzed using SPSS 20.0 software. Count data are reported as frequency (N) and ratio (%). Quantitative data were expressed as mean ± standard deviation (SD). A nonparametric rank-sum test was used to compare data with a skewed distribution, and the quartile distance M (P25, P75) was used. The chi-square test (χ^2^) was used to compare differences across sexes, grades, and majors. Ordinal logistic regression models were constructed to explore the potential influencing factors of anxiety symptoms, depressive symptoms, and sleep quality during the lockdown.

## 3. Result

### 3.1. Participants’ characteristics

Participants’ characteristics are presented in Table 1. Of the 6905 respondents, 3575 (51.77%) were male, and 3330 (48.23%) were female. Of the overall sample, 459 (6.65%) were younger than 18, and 6446 (93.35%) were 18 to 25. There were 4293 (62.17%) freshmen and 2612 (37.83%) sophomores; Regarding academic major,2645 (38.31%) were studying a medical specialty, and 4260 (61.69%) were studying a non-medical specialty. The reported incidences of mental disorders were as follows: sleep disorder 1668 (24.16%), anxiety 419 (6.07%), depression 2984 (43.22%), any of the 3 3045 (44.10%), 2 of the 3 763 (11.05%), and all 3 162 (2.35%).

**Table 1:** Characteristics of the survey respondents from the college (n = 6905).

Variables	Number n (%)
Age (yr)	
<18	459 (6.65%)
18~25	6446 (93.35%)
Sex	
Male	3575 (51.77%)
Female	3330 (48.23%)
Year in college	
1	4293 (62.17%)
2	2612 (37.83%)
Speciality	
Medical	2645 (38.31%)
Nonmedical	4260 (61.69%)
Sleep disorder Anxiety	1668 (24.16%) 419 (6.07%)
Depression	2984 (43.22%)
Any of the 3	3045 (44.10%)
Two of the 3	763 (11.05%)
All 3	162 (2.35%)

### 3.2. Levels of mental health and sleep disorder

Table 2 shows how mental health was affected during school closures. Of the 6905 students, approximately 94% had no symptoms of anxiety; the proportions with mild, moderate, and severe anxiety were 5.65%, 0.36%, and 0.04%, respectively. The incidences of anxiety symptoms were higher in female college students compared to male students (7.6% vs 4.64%) (*P* < .001). Moreover, students with medical majors were more likely to be severely anxious (8.73% vs 4.44%) (*P* < .001). However, grade had no significant effect on anxiety (*P* > .05).

**Table 2:** Number of students with different anxiety levels (n = 6905).

SAS	<50	50~59	60~69	≥70	χ^2^ *P*
Anxiety level	Normal	Mild	Moderate	Severe	
Total participants	6486	391	25	3	
Male Female	3409 3077	158 233	8 17	0 3	χ^2^ = 28.557 *P* < .001
Percentage of males (%)	95.36	4.42	0.22	0	
Percentage of females (%)	92.40	7.00	0.51	0.09	
Percentage of males in total (%)	49.37	2.29	0.12	0	
Percentage of females in total (%)	44.56	3.37	0.25	0.04	
Medical total (%)	2414 (91.27%)	211 (7.98%)	17 (0.64%)	3 (0.11%)	χ^2^ = 55.74 *P* < .001
Nonmedical total (%)	4072 (94.06%)	180 (4.2%)	8 (0.2%)	0 (0.04%)	
Year in college (%)					
1	4038 (94.06%)	236 (5.50%)	17 (0.4%)	2 (0.04%)	χ^2^ = 1.056 *P* = .849
2	2448	155	8	1	

Table 3 shows how depression was affected by school closures. More than half (56.78%) of the students had no symptoms of depression, and the proportion of students with mild, moderate, and severe depression was 36.89%, 6.23%, and 0.10%, respectively. The incidence of anxiety symptoms was significantly higher in female than in male students (45.23% vs 41.56%) (*P* < .001). Moreover, students with medical majors were more likely to have severe depression (47.54% vs 42.11%) (*P* < .001). However, grade had no significant effect on depression (*P* > .05).

**Table 3:** Number of students with different depression levels (n = 6905).

SDS	≤52	53~62	63~72	≥73	χ^2^ *P*
Depression level	Normal	Mild	Moderate	Severe	
Total participants	3921	2547	430	7	
Male	2097	1254	217	7	χ^2^ = 18.27 *P* < .001
Female	1824	1293	213	0	
Percentage of males (%)	58.66	35.08	6.07	0.20	
Percentage of females (%)	54.77	38.83	6.40	0	
Percentage of males in total (%)	30.37	18.16	3.14	0.10	
Percentage of females in total (%)	26.42	18.73	3.08	0	
Medical total (%)	1379 (52.1%)	1091 (41.3%)	175 (6.6%)	0 (0.0%)	χ^2^ = 43.792 *P* < .001
Nonmedical total (%)	2542 (59.7%)	1546 (34.2%)	255 (6.0%)	7 (0.2%)	
Year in college (%)					
1	2454 (57.2%)	1572 (36.6%)	264 (6.1%)	3 (0.1%)	χ^2^ = 1.808 *P* = .617
2	1467 (56.2%)	975 (37.3%)	166 (6.4%)	4 (0.2%)	

Table 4 shows how sleep state was affected during the school closures. Of the 6905 students, approximately 25% had no symptoms of sleep disorder; the proportions of students with mild, moderate, and severe sleep problems were 20.18%, 3.56%, and 0.42%, respectively.

**Table 4:** Number of students with different insomnia levels (n = 6905).

ISI	0~7	8~14	15~21	22~28	χ^2^ *P*
Insomnia level	Normal	Mild	Moderate	Severe	
Total participants	5237	1393	246	29	
Male	2888	554	118	15	χ^2^ = 105.665 *P* < .001
Female	2349	839	128	14	
Percentage of males (%)	80.78	15.50	3.30	0.42	
Percentage of females (%)	70.54	25.20	3.84	0.42	
Percentage of males in total (%)	41.82	8.02	1.71	0.22	
Percentage of females in total (%)	34.02	12.15	1.85	0.20	
Medical total (%)	1874 (70.9%)	650 (24.6%)	106 (4.0%)	15 (0.6%)	χ^2^ = 59.843 *P* < .001
Nonmedical total (%)	3363 (78.9%)	743 (17.4%)	140 (3.3%)	14 (0.3%)	
Year in college (%)					
1	3288 (76.6%)	830 (19.3%)	159 (3.7%)	16 (0.4%)	χ^2^ = 6.041 *P* = .110
2	1949 (74.6%)	563 (21.6%)	87 (3.3%)	13 (0.5%)	

The prevalence of sleep disorders was significantly higher in female than in male students (14.21% vs 9.95%) (*P* < .001). Moreover, students with a medical specialty were more likely to have severe sleep disorders (29.2% vs 21%) (*P* < .001). However, grade had no significant effect on sleep quality (*P* > .05).

Factors influencing anxiety, depressive symptoms, and sleep quality.

The associations between influencing factors and anxiety symptoms, depressive symptoms, and sleep quality during the COVID-19 Omicron outbreak are presented in Tables 5–7. The model test and test of parallel lines indicated a good fit with the observed values (χ^2^ = 10.579, *P* > .05).

The factors influencing the anxiety symptoms are shown in Table 5. The results indicated that female sex (odds ratio (OR) = 1.503, 95% confidence interval (CI): 1.191–1.897) and medical major (OR = 1.367, 95% CI: 1.078–1.734) were risk factors for anxiety. Moreover, compared with sophomores, freshmen had fewer anxiety symptoms (OR = 0.977, 95% CI: 0.782–1.221).

**Table 5:** Ordinal logistic regression analysis of factors influencing junior college students’ anxiety.

Variables	B	OR	*P*	0R (95% CI)
Sex Female Male*	0.408	1.503	.001	1.191–1.897
Speciality Medical Nonmedical*	0.313	1.367	.01	1.078–1.734
Year in college 1 2	0.023	0.977	.838	0.782–1.221

* Control.

Table 6 shows the results of an ordinal regression analysis using COVID-19-related variables as dependent variables, and the survey as independent variables. Male sex (OR = 1.140, 95% CI: 1.023–1.270), non-medical major students (OR = 1.289, 95% CI: 1.148–1.448), and grade 2 students (OR = 1.036, 95% CI: 0.931–1.153) were significant protective factors against depression.

**Table 6:** Ordinal logistic regression analysis of factors influencing junior college students’ depression.

Variables	B	OR	*P*	0R (95% CI)
Sex Female Male*	0.131	1.140	.017	1.023–1.270
Speciality Medical Nonmedical*	0.254	1.289	.001	1.148–1.448
Year in college 1 2*	0.035	1.036	.515	0.931–1.153

* Control.

Table 7 shows the results of the logistic multivariate regression analyses exploring the effects of anxiety, depression, sleep status, sex, and specialty. Medical major (OR = 0.788, 95% CI: 0.661–0.939) was found to be significantly protective against depression. Compared with male students, female students (OR = 2.052, 95% CI: 1.646–2.55) were at a higher risk of sleep disorders. Compared to grade 2 students, grade 1 students were at a higher risk of sleep disorders (OR = 1.157,95% CI: 0.961–1.394).

**Table 7:** Ordinal logistic regression analysis of factors influencing junior college students’ ISI scores.

Variables	B	OR	*P*	0R (95% CI)
Sex Female Male*	0.719	2.052	.001	1.646–2.559
Speciality Medical Nonmedical*	0.239	0.788	.008	0.661–0.939
Year in college 1 2*	0.146	1.157	.124	0.961–1.394

* Control.

## 4. Discussion

This study examined depression, anxiety, and sleep disorders among junior college students in China during school closures during the COVID-19 pandemic. The occurrence rate of the novel coronavirus has led to the total cessation of activities within the examined urban area during the spring of 2022. Consequently, all the college students were limited to online classes. This change in teaching methods has resulted in changes in students’ lifestyles, such as increased sedentary activities and decreased extracurricular and social activities, which may result in increased fatigue, loneliness, anxiety, depression, and sleep disorders.^[21,22]^ Therefore, an online survey was conducted in this study. To the best of our knowledge, this is a cross-sectional big data survey of depression, anxiety, and sleep disorders among junior college students during the COVID-19 lockdown at the study site. Our study included 6905 freshmen and sophomores, and the results showed that the prevalence of psychological and sleep problems was 3045 (44.10%), with the rates of anxiety symptoms, depression symptoms, sleep disorders, 2 of the 3 symptoms, and all 3 symptoms being 1668 (24.16%), 419 (6.07%), 2984 (43.22%), 763 (11.05%), and 162 (2.35%), respectively. Although students’ emotions and sleep conditions are controllable, schools should pay more attention to students experiencing a combination of 2 or even 3 symptoms to avoid aggravation of negative emotions and sleep disorders.

In our study, factors related to anxiety included sleep disorders, depression, sex, and major. Factors related to sleep disorders included anxiety, depression, sex, and major, while factors related to depression included anxiety, sleep disorder, and gender. Anxiety and depression in junior college students during lockdown showed high variability. Only 6.07% of students reported anxiety, but nearly half reported some degree of depression (43.22%).

The prevalence of anxiety and depression symptoms in junior college students during the COVID-19 school closure in our study was lower than that reported in previous studies. For example, an online study of 3092 university students in Nanchang, China, reported that the incidences of anxiety symptoms, sleep problems, either of the 2, and both were 16.8%, 13.5%, 25.1%, and 5.3%, respectively.^[13]^ An online study of 24,678 college students in Henan, China found that 7.3% of respondents experienced anxiety symptoms.^[23]^ Multiple regression analysis revealed that sex, place of residence, and fear levels were related to anxiety after adjusting for confounding factors.^[23]^ An online survey of 1172 residents in 113 cities in Hubei and Guangdong provinces in China showed that the prevalence of anxiety symptoms, depression, and clinical insomnia was 18.8, 13.3, and 7.2%, respectively.^[24]^ A study of 7143 medical college students in China indicated that the prevalence of anxiety symptoms was 24.9%, with mild, moderate, and severe anxiety rates of 21.3%, 2.7%, and 0.9%, respectively.^[25]^ In the USA, an online study of 200 college students found that students closer to graduation faced increased anxiety (60.8%), feelings of loneliness (54.1%), and depression (59.8%).^[12]^ In Greece, an online study of 1000 college students indicated that the prevalence of anxiety symptoms, depression, total suicidal thoughts, and sleep disorders was 42.5%, 74.3%, 63.3%, and 66.3%, respectively.^[26]^ In France, an online study of 3936 students showed that the prevalence of anxiety was 61%, with moderate and severe anxiety rates of 15.2% and 9.8%, respectively. A regression model analysis revealed that being female and having relatives or acquaintances hospitalized for COVID-19 were major risk factors for anxiety^.[27]^ In Saudi Arabia, an online study of 582 undergraduate students indicated that respondents experienced high levels of depression, anxiety, and perceived stress, and low levels of resilience during the pandemic. Additionally, the students reported experiencing insomnia^15^. These results are consistent with our findings. Specifically, female sex was a risk factor for anxiety, depression, and insomnia.

The prevalence rates may vary across studies for the following reasons. First, the samples were not fully representative of those facing isolation. Second, we used scales different from those used in previous studies. Third, the duration of the COVID-19 lockdown varied across the studies. Given that the pandemic has lasted for more than 2 years, most students experienced initial panic, fear, fear, and other negative emotions, and eventually developed a slightly calm mentality. Fourth, national publicity, vaccination rates, government controls, and attitudes toward finding and solving problems in a timely manner can alleviate student anxiety and depression to varying degrees, all of which vary across time and location.

Sleep problems may affect the mental health of college students. In this study, 24.16% of freshmen and sophomores had insomnia due to the COVID-19 pandemic and school closures. Among these students, 3.56% experienced moderate insomnia and 0.42% experienced severe insomnia. Sleep disorders in college students can manifest as difficulty in falling asleep, short sleep maintenance time, waking during the night, lack of sleep, daytime dysfunction, and other forms.^[28]^ The factors related to sleep problems in this study were gender, major, and grade. Compared with male, females were more prone to sleep problems (19.22% vs 29.46%). This could be because females are may spend more time thinking about being unable to go out, have more difficulty staying in confined spaces, and worry about the growing pandemic and the risk to their friends and family, which could easily lead to insomnia. Compared to non-medical majors, medical students were more likely to experience insomnia (40.4% vs 47.9%). This could be because a certain level of medical knowledge comes with a more in-depth understanding of Tcovid-19 and, virus mutations, including Omicron, which is characterized by strong infection, fast transmission, and a higher number of infections. Moreover, medical students are exposed to infected and isolated interns and hospital teachers, which may accelerate the occurrence of sleep disorders and be associated with anxiety and depression. Although the rate of insomnia in grade 2 students was higher than that in grade 1 students, the difference was not statistically significant. This may be because Grade 1 students have recently entered university and may not have fully adjusted to their new living conditions. When this situation is coupled with the impact of the pandemic and restrictions on outdoor activities, sleep problems can develop or become aggravated.

During the pandemic, many headmasters, counselors, teachers, and psychological counselors voluntarily stayed at their schools, communicated online with students daily, helped students with problems in their lives, and helped reduce the negative emotions and sleep problems they experienced in relation to school closure. In physically and mentally safe environments, students are more likely to be able to emotionally regulate and take action to decrease the impact of the pandemic on their well-being.

## 5. Limitations and strengths

This study had several limitations. First, the sample comprised voluntary participants and had limited representativeness, which may have led to a selection bias. Second, the research period was different from that of previous studies, which may be why the results differed from those of previous studies. Furthermore, an important limitation is that we only collected information from junior college students in the target city.

## 6. Conclusions

Global crises such as COVID-19 will undoubtedly cause depression, anxiety, and sleep disorders. Junior college students are more likely to develop mental disorders when faced with such situations. The findings suggest that schools and all sectors of society should strengthen education related to public health emergencies, such as the new coronavirus, improve students’ cognitive level, and encourage and help individuals carry out regular physical and recreational activities, which can reduce the risk of depression, anxiety, and sleep disorder problems and help junior students maintain the best physical condition.

## Acknowledgments

The authors express their sincere appreciation to all the people involved in the study and their partner schools.

## Author contributions

**Conceptualization:** Wenxia Hu.

**Data curation:** Feng Li.

**Formal analysis:** Shouliang Ma.

**Investigation:** Feng Li, Jing Wang, Shouliang Ma.

**Supervision:** Jiu Chen, Maoxue Wang, Wenxia Hu.

**Software:** Maoxue Wang.

**Writing – original draft:** Feng Li.

**Writing – review & editing:** Bing Zhang, Jiu Chen, Qian Chen, Junxia Wang, Maoxue Wang.
